# Biological properties of calcium phosphate biomaterials for bone repair: a review

**DOI:** 10.1039/c7ra11278e

**Published:** 2018-01-09

**Authors:** Jingyi Lu, Huijun Yu, Chuanzhong Chen

**Affiliations:** Shenzhen Research Institute of Shandong University Shenzhen 518057 Guangdong P. R. China yhj2001@sdu.edu.cn czchen@sdu.edu.cn +86 531 88395991 +86 531 88395991; Key Laboratory of High-Efficiency and Clean Mechanical Manufacture (Shandong University), Ministry of Education, School of Mechanical Engineering, Shandong University Ji'nan 250061 Shandong P. R. China; National Demonstration Center for Experimental Mechanical Engineering Education (Shandong University), School of Mechanical Engineering, Shandong University Ji'nan 250061 Shandong P. R. China; Key Laboratory for Liquid-Solid Structural Evolution and Processing of Materials (Ministry of Education), School of Materials Science and Engineering, Shandong University Ji'nan 250061 Shandong P. R. China

## Abstract

Bone defects are a common disease threatening the health of many people. Calcium phosphate (CaP) is an ideal bone substitutive material that is widely used for bone repair due to its excellent biological properties including osteoinductivity, osteoconductivity and biodegradability. For this reason, investigation of these properties and the effects of various influencing factors is vital for modulating calcium phosphate during the design process to maximally satisfy clinical requirements. In this study, the latest studies on the biological properties of CaP biomaterials, including hydroxyapatite (HA), tricalcium phosphate (TCP), and biphasic calcium phosphate (BCP), have been summarized. Moreover, recent advances on how these properties are altered by different factors are reviewed. Considering the limited mechanical strength of CaP materials, this study also reviews CaP composites with different materials as improvement measures. Finally, perspectives regarding future developments of CaP materials are also provided.

## Abstract 2

This article reviews the recent advances and various factors affecting the improvement of the biological properties of calcium phosphate for bone repair.

## Graphical abstract

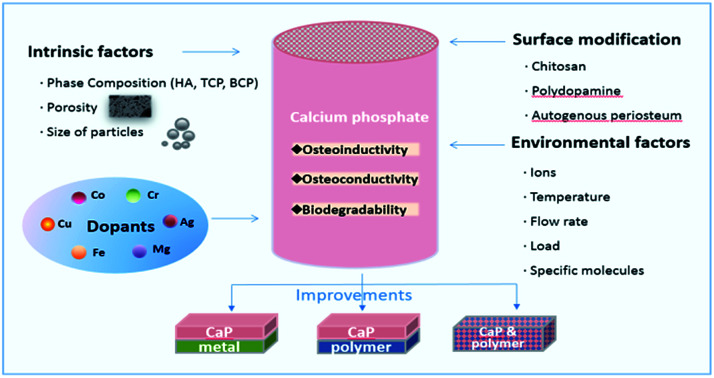

## 1. Introduction

Nowadays, bone defects are among the most common diseases in clinical orthopedics and are mainly caused by infections, defects, tumors, and congenital diseases.^1^ Generally, these defects need bone grafts since they cannot heal by themselves, and inappropriate treatment can lead to death or invalidity.^2^ Researchers have, therefore, tried to find novel materials for bone repair and substitution.

Autologous bone is still the most commonly used bone material, but it is accompanied by some limitations such as requiring additional surgical incisions.^3,4^ Even though the allograft or heterogeneous bone has a richer source than autogenous bone, it lacks osteogenetic potential and may be rejected or may spread disease and are therefore not ideal candidates for bone repair.^5^ Moreover, metal scaffolds and polymeric materials lack bioactivity, even if they have shown great potential regarding mechanical properties.^4,6^ In magnesium implants, for example, the rapid degradation rate *in vivo* has become the main limitation hindering their applications;^7^ thus, they cannot be considered as prominent candidates for use in bone reconstruction.

Recently, calcium phosphate materials have been gradually attracting significant attention due to their excellent abilities to induce osteoblast differentiation into bone cells,^8,9^ causing them to grow on the surface of scaffolds^10,11^ and degrade at proper rates,^12,13^ and even be totally replaced by newly formed bone tissue, in addition to withstanding stress at the defect site.^14^ Within the calcium phosphate family, HA (hydroxyapatite) has been widely studied since the 1980s,^15^ and its inorganic composition is similar to that of natural bone. The crystalline network of the stoichiometric HA can be described as a compact assemblage of tetrahedral PO_4_ groups, where P^5+^ ions are in the center of the tetrahedrons and whose tops are occupied by 4 oxygen atoms. Each PO_4_ tetrahedron is shared by a column and delimits two types of unconnected channels.^16^ The most common ways to prepare HA are by the solution-precipitation method^17–19^ and the sol–gel method.^20,21^ Endowed with a composition and appropriate porosity similar to natural bone, HA exhibits great osteoconductivity and vasculogenesis, thus serving as an outstanding material for achieving strong bonds with the host bone, and prompting vascular formation; however, its poor mechanical properties hinder its applications in load-bearing circumstances.

As another extensively studied form of calcium phosphate, tricalcium phosphate (TCP) has three polycrystalline morphologies: α, β and ά, which exist at different temperatures and exhibit great bioactivity as well as high degradability.^22^ ά lacks practical interest because it only exists at temperatures >1430 °C and reverts almost instantaneously to α-TCP on cooling below the transition temperature. In contrast, β-TCP is stable at room temperature and transforms reconstructively at ∼1125 °C to α-TCP, which can be retained during cooling to room temperature.^23^ α-TCP has better solubility than β-TCP in aqueous solutions^24^ and it is not commonly used for the preparation of bioceramics, but is used in the preparations of calcium phosphate bone cement and calcium phosphate composite ceramics.^25^ β-TCP is a metastable phase that occurs during the cooling process, which shows high stability at room temperature and has excellent bioactivity properties, especially in inducing apatite deposition.^23^ Jarcho *et al.*^26^ reported that the solubility of TCP is 22.3 times that of HA in alkaline solution. As for mechanical properties, they can be altered by various factors such as porosity, sintering temperature and the shape of the CaP scaffold; for example, the compressive strength of the HTCP (hierarchically porous tricalcium phosphate) scaffold is 5.06 ± 1.21 MPa,^27^ whereas a porous TCP with porosities of 82% and 49% are 0.4 MPa and 40 MPa, respectively.^28^ To make it clear, some major properties of HA and TCP are reviewed in Table 1.

**Table 1:** HA, TCP and their major properties^29,30^

Material	Stoichiometry	Crystallography	Ca/P ratio	Solubility at 25 °C	
				−log *K*_s_	g l^−1^
HA	Ca_10_(PO_4_)_6_(OH)_2_	Hexagonal	1.67	116.8	0.00010
β-TCP	β-Ca_3_(PO_4_)_2_	Rhombohedral	1.50	28.9	0.20
α-TCP	α-Ca_3_(PO_4_)_2_	Monoclinic	1.50	25.5	0.97
ά-TCP	ά-Ca_3_(PO_4_)_2_	Hexagonal	1.50	25.5	0.97

There is another important type of calcium phosphate called biphasic calcium phosphate (BCP), consisting of HA and TCP. Due to the advantages of BCP achieved by adjusting the ratio of HA/TCP, the degradation rate can be modified to match the degree of new bone formation.^31,32^

It is important for researchers to investigate the biological abilities of scaffolds, since both the general and detailed conditions of bone repair are reflected by osteogenic activity.^8^ One of the most crucial biological properties is osteoinductivity, which is normally used to demonstrate the ability of the implant to induce bone growth in an ectopic site. The mechanism of inducing osteogenesis is that Ca and P ions existing in body fluids gather on the surface of the implant and form an ion layer to integrate with proteins close to the bone cells; therefore, the implant material is able to firmly connect with the host through this ion layer.^33^ In this case, a deep investigation of the interaction between the material surface and various proteins and cells can lead us to a better understanding of the osteoinduction process as well as its requirements for an ideal biomaterial. Unlike osteoinduction, osteoconduction is a phenomenon where after bioactive materials are implanted into the bone environment, bone tissue will grow along the surface or internal pore of the implant. Therefore, proper porosity is desirable for achieving great osteoconductivity by providing space for cells to crawl and grow through the channel inside the scaffold. Osteoconductivity is usually reflected in bone coverage measurement, which can be achieved by SEM analysis and micro-computed tomography.^34^ Biodegradability refers to the biological property of a material dissolving with time after implantation into the body, accompanied by the decrease of mechanical properties of the implanted materials.^35^ So far, most of the research has concentrated on understanding how the physiological environment changes the material. It is equally important to understand how the surrounding cells react to the degrading material.

Researchers are able to modulate the performance of bone substitute material by studying the influence of various factors on these biological properties. For instance, different porosities of calcium phosphate scaffolds lead to various degrees of healing of segmental bone,^36,37^ degradation of materials^38^ and drug release kinetics.^39^ The optimization of the phase composition is thought to improve the osteoinductivity of the CaP ceramics, thus helping the restoration of the bone defect. In this case, a combination of the influences of the above factors is usually necessary, as is an assessment of whether each factor needs to be analyzed separately, or if a combined analysis is reasonable.

The factors that considerably influence the biological properties of CaP materials have not been systematically investigated in previous reports. Therefore, in this paper we discuss several important biological properties including osteoinduction, osteoconduction and biodegradation of calcium phosphate materials as well as their influencing factors from several aspects. Some improvements made by researchers to overcome the limitations of CaP in their mechanical properties are also reviewed.

## 2. Biological properties of calcium phosphate biomaterials

Calcium phosphate biomaterials are attracting attention in the field of bone regeneration, particularly due to their excellent biological properties. As aforementioned, the highly complex environment of the body requires that calcium phosphate biomaterial must be able to degrade at a suitable rate to achieve a balance with the regeneration of new bone tissue, and simultaneously provide an ideal place for cell growth. Generally, there are three main properties of calcium phosphate materials that have been widely studied by researchers.

### 2.1. Osteoinductivity

Osteoinduction implies the recruitment of immature cells and the stimulation of these cells to develop into preosteoblasts. In a bone healing situation such as that from a fracture, the majority of bone healing is dependent on osteoinduction.^40^

Interestingly, some osteogenic agents including TGB-β, BMPs, Wnt and other growth factors *via* related signaling pathways are fundamentally important throughout this osteoinduction process. They exhibit versatile regulatory functions in modulating signal transduction, gene expression and osteoblastic differentiation of various cells.

Transforming growth factor-beta (TGF-β)/bone morphogenic protein (BMP) signaling is involved in the vast majority of cellular processes and is fundamentally important throughout life. TGF-β/BMPs have widely recognized roles in bone formation during mammalian development and exhibit versatile regulatory functions in the body.^41^

**Fig. 1:**
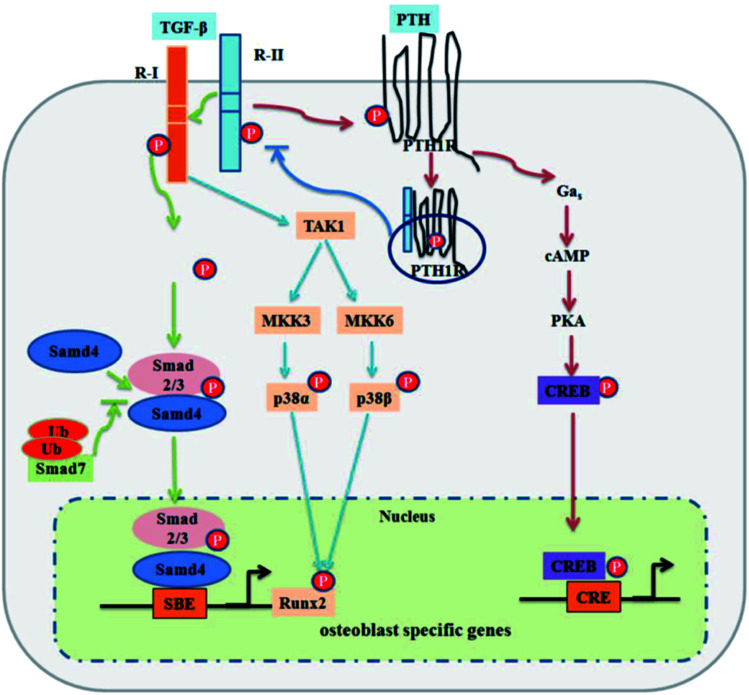
TGF-β signaling and negative regulation in bone formation. Reprinted with permission from ref. 41 (Copyright © 2012, IVYSPRING INTERNATIONAL PUBLISHER).

The transforming growth factor-beta (TGF-β) superfamily is comprised of over forty members, such as TGF-βs, nodal, activin, and bone morphogenetic proteins (BMPs). TGF-β signaling first transmits signals across the plasma membrane through the formation of heteromeric complexes of specific type I and type II serine/threonine kinase receptors. The type I receptor is phosphorylated following the activation of specific type II receptors. Activated type I receptors initiate intracellular signaling through phosphorylation of specific Smad proteins, R-Smads. Activated R-Smads form a complex with co-Smad and Smad4 and then translocate into the nucleus to direct the transcriptional response (Fig. 1).^42^

**Fig. 2:**
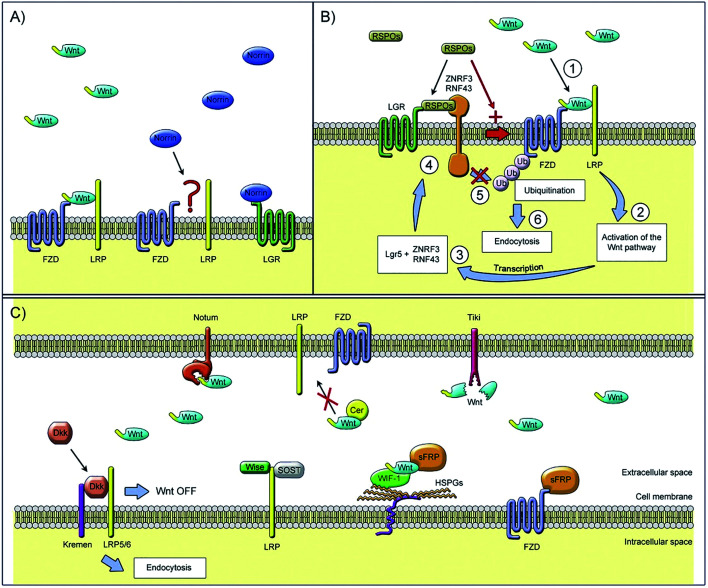
Extracellular regulators of the Wnt signaling pathway: (A) Wnt ligands use diverse co-receptors to activate and modulate different downstream signals in the Wnt signaling pathway. (B) After the binding of Wnt ligands to the frizzled receptor and LRPs co-receptors, Wnt signaling is activated and causes the transcription of gene targets. (C) Models of Wnt signaling inhibition. Reprinted with permission from ref. 46 (Copyright © 2017, Elsevier).

There is another signal molecule called Wnt, which is capable of modulating new bone formation alone or synergistically with BMP *via* its two kinds of signaling pathways: canonical and noncanonical pathways (signaling pathway can be seen in Fig. 2).^43^ Wnts are a family of secreted glycoproteins that regulate many processes in skeletal development. Wnt proteins bind to their cognate receptor frizzled (Fz) and LRP-5/6 co-receptors, and activate distinct signaling pathways, including the canonical β-catenin pathway. In the absence of Wnt signaling, β-catenin is degraded by the proteasome system after GSK3β dependent phosphorylation. In the presence of Wnt signaling, unphosphorylated β-catenin accumulates in the cytoplasm and translocates into the nucleus where it associates with Tcf/LEF transcription factors to regulate the expression of target genes.^44^ Canonical Wnt signaling stabilizes nuclear β-catenin levels and targets gene activation, whereas non-canonical Wnt signaling activates c-Jun N-terminal kinase (JNK) or calcium/calmodulin-dependent kinase 2, which results in convergent extension movements and cellular polarization.^45,46^

Many studies have demonstrated the vital effect of the Wnt signaling pathway on osteogenesis.^47,48^ For example, the deletion of mouse β-catenin in early mesenchymal precursors results in the loss of Runx2 and Sp7 expression and a corresponding failure of bone formation, suggesting that osteogenesis requires Wnt/β-catenin signaling.^49^ According to Okamoto *et al.*,^50^ Wnt5a-induced noncanonical signaling cooperates with Wnt/beta-catenin signaling to achieve proper bone formation, since noncanonical Wnt5a is proved to enhance Wnt/beta-catenin signaling during osteoblastogenesis. Besides, according to Zhang *et al.*,^44^ BMP9 and Wnt3A may act synergistically to induce osteo/odontoblastic differentiation of stem cells of dental apical papilla (SCAPs). Using an *in vivo* stem cell implantation assay, they found that while BMP9-transduced SCAPs induce robust ectopic bone formation, SCAPs stimulated with both BMP9 and Wnt3A exhibit more mature and highly mineralized trabecular bone formation. It is conceivable that TGF-β, BMPs and/or Wnt3A may be explored as efficacious biofactors for odontogenic regeneration and tooth engineering.

Wnt3a reduced osteoclast formation when applied to early bone-marrow macrophage (BMM) osteoclast differentiation cultures, whereas late addition did not suppress osteoclast formation. Results indicate that Wnt3a directly suppresses osteoclast differentiation through both canonical (β-catenin) and noncanonical (cAMP/PKA) pathways in osteoclast precursors, osteoblast numbers as well as trabecular bone mass.^51^ This is opposite to the conclusion of previously published studies that the Wnt signaling pathway promotes osteoblastic proliferation.^52^ Thus, the specific mechanism related to the complexity of various signaling pathways still needs to be explored.

Unlike most metallic materials lacking osteogenesis ability, some calcium phosphate ceramics have been reported to induce bone formation in ectopic sites without adding any more osteogenic agents (*e.g.*, TGF, BMPs or Wnt) to the material. This phenomenon is called “osteoinduction,” and the capacity for osteoinduction is called “osteoinductivity” or “osteoinductive potential”. Normally, whether a material is bone induced depends on whether it can still function well, even in the situation of ectopic implantation such as subcutaneous injection^53^ and intramuscular implantation.^54^

**Fig. 3:**
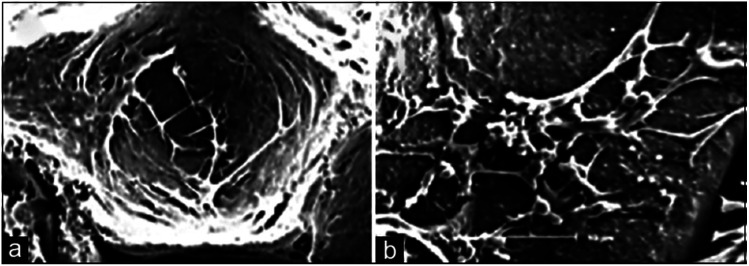
Scanning electron microscopy technique: (a) stem cells adhered in the pores of the scaffold and (b) extracellular matrix as the deposition of granular products secreted by differentiated osteoblasts in the pores of the hydroxyapatite/tricalcium phosphate scaffold. Reprinted with permission from ref. 59 (Copyright © 2016, Wolters Kluwer India Pvt. Ltd).

Among numerous biomaterials, calcium phosphate materials, such as HA, TCP and BCP have been regarded as prominent candidates for bone reconstruction due to their great osteoinductivity, which is of importance for the adsorption and differentiation of mesenchymal cells into osteoblasts and osteocytes, further enhancing the vascular formation as well as bone tissue regeneration.^55,56^ With osteoinductive ability, these CaP ceramics were able to achieve better bone regeneration *in vivo*, even in a critical size defect, without the addition of cells or growth factors.^5^ Implanting porous nano-crystalline HA into the back region of mini pigs^18^ was found to result in a phenomenon of ectopic osteogenesis characterized by the formation of bone-like structures and tendon-like structures with bone marrow and focal chondrogenesis; bone formation was better in the subcutaneous than in the intramuscular implantation sites.^57^ TCP microspheres have excellent bioactivity properties, specifically in inducing apatite deposition,^22^ which can achieve selective absorption of serum protein so as to enhance the adhesion of osteoblasts and the secretion of collagen fibers.^33,58^ The BCP scaffold can provide a suitable environment for the differentiation and activity of osteoblasts according to the extracellular matrix secreted by osteoblasts, observed in the pores of hydroxyapatite/tricalcium phosphate scaffold, which can be seen as an important marker for osteogenesis induction (Fig. 3).^59^

Based on the extensive research work surrounding these biomaterials, the intriguing phenomena have been categorized as those influenced by material factors, and those by biological factors. It is well known that osteoinduction is highly dependent on several material parameters including porosity, granule size, phase composition and even sintering temperature, which will be discussed in Section 3.

As for biological factors, the osteoinduction of calcium phosphate reportedly varies with different animals including dogs, mice, rabbits and pigs. Interestingly, the same material may exhibit discrepancies on different animals. Cheng *et al.*^14^ compared the osteoinductivity of calcium phosphate ceramics in four kinds of animals. Results showed that CaP ceramics have good biocompatibility and biological safety, and the degree of ease of osteogenesis was as follows: mouse > dog > rabbit > rat.

Based on conditions mentioned before, graft materials played a significant role in inducing bone cells to grow and differentiate, but the mechanism of osteoinduction was not well explored, including the source of stem cells, the type of signaling molecules and transforming factors, since most of the molecular biotechniques were only based on rodents.^8^ Thus, it is essential for researchers to focus their studies of osteoinduction assessment on large animals, continuing to detail the mechanisms of osteoinduction on a molecular level.

### 2.2. Osteoconductibility

There is a phenomenon where after implanting bioactive materials into a bone environment, bone tissue will grow along the surface or internal pore of the implant, which is called bone conduction.

The most common and effective way to characterize the osteoconductivity of biomaterials is using bone coverage measurement, which can be induced from SEM images and biopsy analysis including micro-computed tomography, non-calcified histology and histomorphometry.^34^ The more tissue and bone cells that are grown on substitute implants, the better will be the osteoconductivity.

Recently, Fern *et al.*^60^ established a constitutive equation that is able to simulate the osteoconduction model based on a numerical analysis. This reaction–diffusion equation incorporates mass matrix, osteogenic cells and osteoblasts; growth factors considering these variables are closely related to multiphysics interactions associated with the bone-implant interface. Let *m*, *S*_2_ ∈ *R* be the density of osteogenic cells and the concentration of osteogenic growth factors (BMPs, TGB-β), respectively. For the density of the osteogenic cells, *m*, the flux is modelled with a linear diffusion term and a linear chemotaxis term along the gradient of the growth factor *S*_2_; the kinetics is represented by a proliferative term consisting of a logistic growth with a natural linear rate, and there is a linear term related to the differentiation into osteoblasts and natural cell death such that
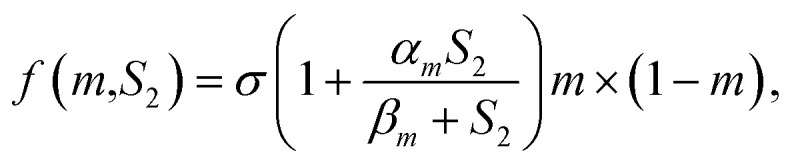
in which *σ* denotes the linear rate coefficient of the proliferative term. Jose *et al.* have also presented one-dimensional numerical simulations to show the accuracy of the approximation and the behavior of the solution. The results suggest that this mathematical approach could be a useful tool that allows improvements of the implant design.^60^

**Fig. 4:**
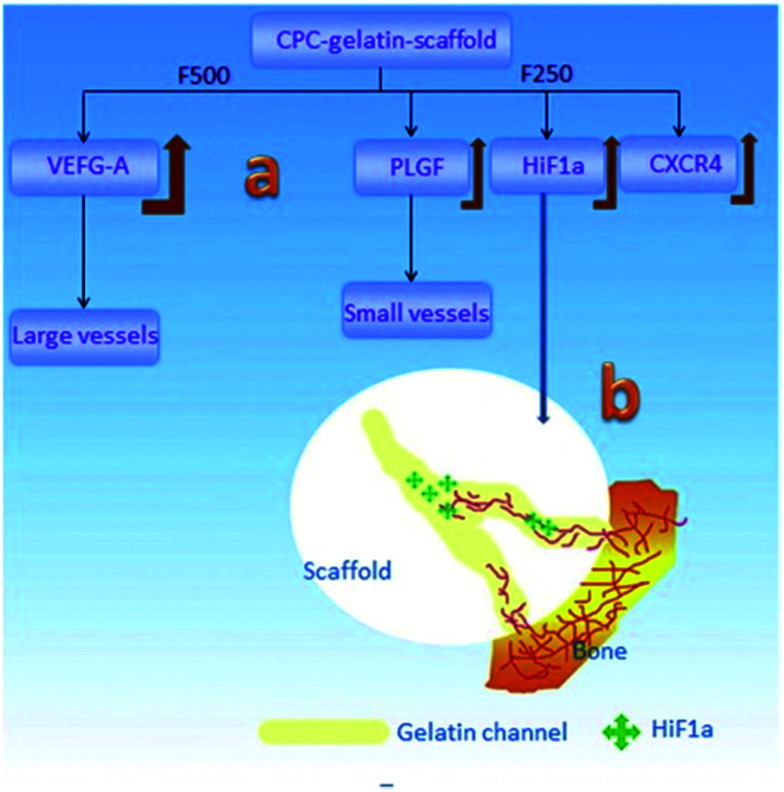
Schematic of the vascularization strategy within the channels of the CaP scaffold: (a) different channel diameters induced different expression behaviors for growth factors and then induced the different vessel formation; (b) the gradually-increased HIF1α expression in the channels induced the in-growth of blood vessels into its host. Reprinted with permission from ref. 62 (Copyright © 2016, Elsevier).

Various factors have effects on this osteoconduction process. As mentioned in Section 3.1, osteoconduction is influenced by material-dependent factors as well as the conditions of defect sites. For instance, bone conduction is seen as a characteristic of biomaterials that are not regarded as ideal materials from the point of view of biocompatibility, *e.g.*, stainless steel and titanium,^61^ but as biomaterials having the ability to acquire a particular morphology with proper inner structure and toughness to provide ideal placement for cell events. According to the results of Yu *et al.*,^62^ different channel sizes induce different vascularization (Fig. 4): in the porous CaP scaffold, the channel with the size of 250 pm increases the expression of the representative angiogenic factors HIF1 alpha (hypoxia-inducible factor 1), PLGF (placental growth factor) and migration factor CXCR4 (C-X-C chemokine receptor type 4), which promote the formation of small vessels, while the channel with the size of 500 pm enhances VEGF-A (vascular endothelial growth factor A) expression, which benefits the development of large vessels. Not only does the size of the interconnecting channels have an obvious effect on tissue formation, but it is thought that macroporosity (pores > 50 μm) determines cell colonization and accordingly, the growth of vascular and bone tissue, while the microporosity (<50 μm) of bioceramics increases its protein adsorption, which can in turn determine cell fate.

On the other hand, bone growth depends on the conditions of the defect sites, such as the action of differentiated bone cells, which may originate in osteoblasts activated by trauma, or in cells recruited from primitive mesenchymal cells by osteoinduction.^63^ As suggested in the results of Johari *et al.*,^64^ who prepared a cell-seeded scaffold for the repair of calvarial bone with defects, and a blank scaffold as comparison, the number of new cells stained by H&E (hematoxylin and eosin) in cell-seeded grafts was significantly higher than the defect filled with blank scaffold.

Results have shown that calcium phosphate appears to have the advantages of good osteoconductivity and has been investigated in the clinical treatment of rabbits,^65,66^ dogs^11,62,67,68^ and rats,^69,70^ Yu *et al.*^62^ implanted CaP ceramics with interconnected channels into the defect sites of rats; the results indicated that the in-growth of blood vessels was observed in the border of the scaffold. nHA-coated BCP ceramics seeded with mesenchymal stem cells (MSCs) were prepared and shown to enhance the formation of new bone tissue in the BCP ceramics after being implanted into rabbits for 12 weeks. Based on the references collected in this paper, it can be concluded that calcium phosphate functions well in human patients. According to Friedmann *et al.*,^71^ five patients benefited from three augmentation regimens, the percentage of bone coverage of graft particles for all biopsies ranged from 27.83% to 80.17% (average at 55.39%), which indicated that a close contact between the graft particles and newly formed bone had been achieved. Yang *et al.*^72^ demonstrated that the metal implant coated with the CaP layer promotes migration of osteoprogenitor cells along its surface, and thus accelerates osteogenesis in relation to implants so coated. Still, only a few studies have been reported about *in vivo* osteoconduction of CaP used for human patients, thus, CaP osteoconductibility in human cases still needs to be assessed and characterized with suitable approaches.

### 2.3. Biodegradability

Biodegradable material will dissolve with time after being implanted in the body, accompanying a decrease in mechanical properties of the implanted materials; the loads will gradually transfer from the implants to human bones and soft tissues to avoid the stress shielding effect.

There are two kinds of explanations for the mechanism of calcium phosphate biodegradability, namely, “dispersing material into particles” and “dissolving material into ions”. The former concept is on the basis that implanted material is first dispersed into tiny particles or debris until the debris is transferred by phagocytes or osteoclasts;^73^ the latter concept is based on the premise that the implanted material dissolves and releases Ca^2+^ and HPO_4_^2−^, which are then absorbed by the cells for bone repair and reconstruction, and the formation of new bone.^74^ Subsequently, it was found that these two kinds of mechanisms seem to be able to coexist in the process of osteogenesis, and may be applied to different conditions according to different environments and materials.

Sheikh *et al.*^75^ summarized the degradation process of implanted materials as three reactions (physical, chemical, biological response) and two stages (stage 1: early dissolution; stage 2: cell-mediated absorption).

The physical reaction is the process of degradation *via* dissolving of materials, where the biomaterial surface transforms into bone-like apatite through dissolving, deposition, ion exchange and a series of reactions before the material finally collapses into tiny particles; during this period, mechanical strength and density remarkably decrease.

**Fig. 5:**
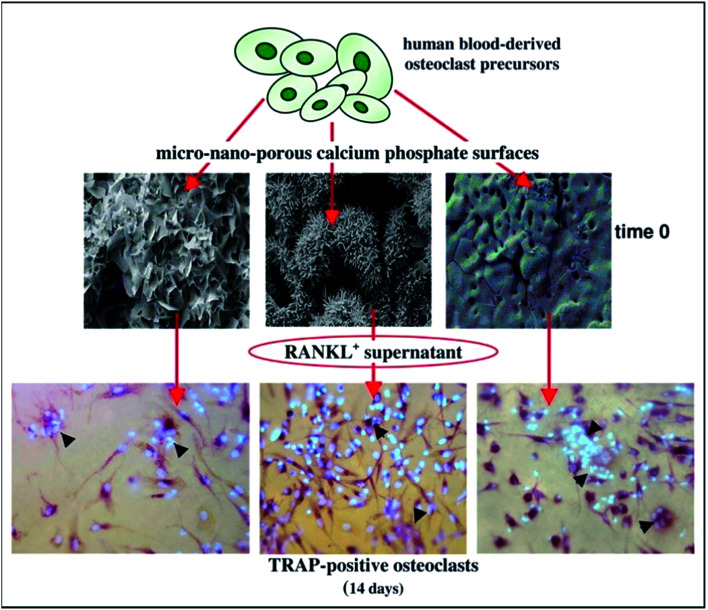
Osteoclasts function on the surface of CaP. Reprinted with permission from ref. 80 (Copyright © 2017, Elsevier).

For the biological response, the degradation and absorption of material involve the mutual participation of cells including osteoclasts,^73,76,77^ osteoblasts, fibroblasts, macrophages^78^ and multinucleated giant cells (Fig. 5).^79^ During bone injury, monocytes and macrophages play diverse roles in repair, modulating the acute inflammatory response, producing growth factors such as BMP-2 and PDGF-BB, and inducing osteogenesis of mesenchymal progenitor cells. Osteoclasts are giant multinucleated bone-resorbing cells differentiated from precursors of the monocyte/macrophage lineage. Their bone resorbing activity is essential for controlling bone development as well as calcium homeostasis.^77^ Osteoblasts and fibroblasts are generally employed after receiving the growing factors released by monocytes/macrophages, and play a significant role in forming new bone tissue.^59,80^

Two important properties of an ideal bioactive bone substitution biomaterial are reportedly thought to be exhibiting the same biomechanical competence and regenerating in the same fashion as autologous bone.^73^ This requires materials to degrade at the same speed at which osteoblasts lay down new bone on their surfaces, until the material is completely replaced by new, living bone.^81^ Ideally, controlled degradation of a biomaterial leads to the consecutive loss of the mechanical strength of the device, which in turn leads to slowly rising forces in the healing tissue, thereby enhancing the healing process and avoiding the unwanted consequences such as stress shielding.

Many biomaterials have been investigated to prove whether they can be employed as absorbable implants. Numerous metals and polymers^35^ cannot degrade properly with time after implantation, while calcium phosphate, especially TCP, has excellent biodegradability. It has been reported that soaking the calcium phosphate cement in DMEM (Dulbecco's minimum essential medium) for an hour results in the precipitated concentration of phosphate ions peaking at (4.380 ± 0.019) mmol L^−1^, calcium ions increase slowly to (1.690 ± 0.064) mmol L^−1^ after 6 h. According to the law of cell proliferation, only when the concentrations of phosphorus and calcium ions become stable at a certain value can the proliferation of cells reach the best state. Thus, controlling the solubility of the substitute bone material in the body is vital both for bone repair and strength enhancement.

Additionally, Wang *et al.*^82^ recently confirmed the correlation between the biodegradation and osteoinductive capacity of BCP ceramic. The ceramic itself and its degradation products can induce macrophages to express and secrete various signaling molecules (TNF-α, IL-6, MCP-1, MCP-1a, MCP-1b and MDC), which then recruit and promote the MSCs to differentiate into osteoblasts. Other studies also mentioned this relation. For example, Wang *et al.*^83^ found that the pro-inflammatory cytokines including TNF-α and IL-6 were less expressed and the bone repair related cytokine of TGF-β1 was up-regulated by macrophages in MCPC (magnesium–calcium phosphate cement) extract. However, there are few detailed mechanisms and much complexity between osteoinduction and biodegradation, which need to be addressed in the future in order to design promising scaffolds for bone repair.

## 3. The factors influencing biological properties

The biological performance of calcium phosphate bioceramics varies with the nature of the material itself, the processing technology and the changes in the external environment. The most important impact on the biological properties of calcium phosphate bioceramics is caused by the intrinsic properties of the material, which are relatively easy to measure and analyse, according to a large number of relevant reports available for study. The influence of the external environment is more complicated, since fluid composition is too complex to control. Given that studying the effect of a particular component usually causes changes in the other ingredients, the effects of the external factors on the properties of calcium phosphate have not been systematically studied.

### 3.1. Intrinsic factors

#### 3.1.1. Phase composition

The composition of the biomaterial has great effects on its performance, such as the compressive strength, crystallinity, mineralization and thermostability, thus significantly influencing the biological properties of the material. As a main factor, how the Ca/P ratio modulates the properties of calcium phosphate ceramics or cement has been investigated by numerous researchers.

The CaP phase always varies with its Ca/P ratio. Zhang *et al.*^84^ obtained calcium phosphate cement with different Ca/P molar ratios (1.50, 1.60, 1.67, 1.80) by adding CaCl_2_ to increase the concentration of Ca in the calcium phosphate cement. Ergun *et al.*^85^ found that for the samples with Ca/P ratios of 0.5 and 0.75, TCP and Ca_2_P_2_O_7_ phases were observed, while those with ratios of 1.5 and 1.6 had TCP and HA, respectively, as their dominant phases. In terms of the BCP samples, HA50TCP50 and HA100TCP0 have Ca/P ratios of 1.59 and 1.67, respectively.^86^ Moreover, the experiment done by Ergun *et al.*^85^ showed that higher Ca/P ratios (up to 2.5) could enhance increased osteoblast adhesion on calcium phosphate, and the interactions of human bone marrow mesenchymal stem cells (BMSCs) with the Ca–P samples.

The optimization of the phase composition is thought to improve the osteoinductivity and other biological abilities of CaP ceramics, thus helping the restoration of the bone defect. Wang *et al.*^87^ found that the group of BCP with 30% HA and 70% TCP promoted the highest expression of BMP-2 and then showed the strongest osteoinduction in mice, compared to several other groups with different phase compositions. HA50TCP50 (Ca/P ratio at 1.59) markedly enhanced cell spreading, proliferation and expression of the extracellular matrix (ECM) genes such as α-smooth muscle actin (α-SMA) and fibronectin (FN), compared with HA100TCP0 (Ca/P = 1.67).^86^ It was also reported that the ceramics with BCP (HA/β-TCP = 9/1) can cause bone-like apatite to form in shorter immersion time, and with the increase of the β-TCP amount, the bone-like apatite formation is easier.^88^ Besides, it was demonstrated that the degradation rate of the carrier and the drug release kinetics could be made tunable within the time scale of 1–2 h for the most soluble CaP phase, monocalcium phosphate (Ca(H_2_PO_4_)_2_), compared to 1–2 years for the least soluble one, HA. From the standpoint of antibiotic therapy for osteomyelitis, typically lasting for 6 weeks, the most promising CaP powder was amorphous CaP.^89^ Similarly, Chen *et al.*^90^ employed amorphous CaP as an effective carrier, loading it with IgY molecules (chicken immunoglobulin Y), which indicated the potential applications in dental care, especially in the prevention and treatment of dental caries. Results showed that the nanospheres exhibited significant antibacterial activity against *Streptococcus mutans* (*S. mutans*), and the as-prepared ACP nanospheres showed a relatively high IgY protein adsorption ability and sustained release behavior.

#### 3.1.2. Porosity

Generally, the porosity is necessary for achieving excellent bone repair, since the inner pores provide a larger surface area, which is believed to contribute to higher bone induction, protein adsorption, as well as ion exchange and bone-like apatite formation by dissolution and reprecipitation.^38,39^

A common way to obtain a porous structure in calcium phosphate is by the sacrificial template method. During this method, slurry is absorbed into a porous template (such as polyurethane sponge^91–93^ or micron/nano-sized graphite^94^) with desired porosity, then it is dried into a solid template for a certain time, followed by sintering the template until it is burnt away, leaving the desired porous structure.

**Fig. 6:**
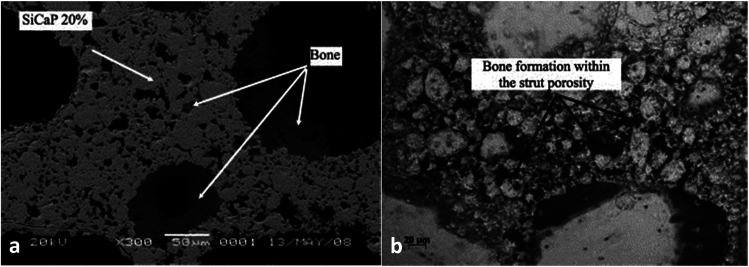
A scanning electron micrograph showing bone formation within (a) the SiCaP-20 scaffold; (b) the SiCaP-30 scaffold. Reprinted with permission from ref. 97 (Copyright © 2012, John Wiley and Sons).

Calcium phosphate porosity plays a significant role in modulating its biological properties including degradation, osteoconductivity and osteoinductivity. Woodard *et al.*^95^ suggested that the cause of a greater decrease in the strength of MP scaffolds (CaP with microporosity and macroporosity) after implantation, in comparison to the NMP (CaP only with macroporosity) scaffolds may be the greater osteoclast-induced degradation at the chemically reactive grain boundaries than that of NMP. Kasuya *et al.*^96^ prepared CPC (calcium phosphate ceramic) mixed with gelatin powder to create different porosities at 10% (C10) and 15% (C15) respectively, and the residual composite area was observed to decrease from 65% to 31% in C10, and 70% to 20% in C15, which indicated that the degradation degree of CPC was positively related to the porosity. Besides, it has been demonstrated that the large specific surface area, which can be achieved by increasing the number of micropores, is essential for osteoconductivity for bone regeneration. According to Woodard *et al.*,^95^ bone was formed only in the CaP scaffolds containing microporosity, demonstrating superior osteoconductivity compared to those without. This can be attributed to microporosity-improved growth factor retention, upon which bone formation largely depends in ectopic sites. Similarly, the group HA-nG-25% that contained more micropores was possibly more favorable for allowing the full in-growth of cells.^94^ In general, the nanoporosity in scaffolds could significantly promote osteoinductivity during bone tissue engineering by enhancing osteogenic differentiation.^94^ The greater bone formation was seen in scaffolds with increased strut porosity (from 20% to 30%) in an ovine ectopic model (Fig. 6).^97^ Generally, microporosity is necessary to achieve excellent osteoinductivity resulting from inner pores providing a larger surface area, which is believed to contribute to higher bone induction and protein adsorption as well as ion exchange and bone-like apatite formation by dissolution and reprecipitation.^38,39^ Tsukanaka *et al.*^98^ observed osteoinduction in half of the β-TCP materials with 60% porosity implanted into mice, whereas there was no osteoinduction in the 75% porosity group, although the latter exhibited the greatest number of vessels.

Localized drug/growth factor release requires the biomaterials to have permeable porous structures as scaffolds and carriers. The CaP composite prepared by Yang *et al.*^99^ had a unique three-dimensional structure with interconnected nanopores and exhibits liquid permeability and absorbability.

On the other hand, the increase in porosity will sacrifice the mechanical strength. The BCP powders (60% HA, 40% β-TCP) obtained by Kim *et al.*^100^ showed that with the porosity increasing from 43.0% to 45.9%, the compressive strength decreased accordingly (46.8 to 33.1 MPa). As such, further studies are needed to focus on the relationship between porosity and the performance of calcium phosphate materials, and to investigate the optimal porosity for bone regeneration.

It is worth mentioning that element concentration may have notable effects on the microstructure and porosity of composite biomaterials. For instance, in CaAl–CaP composites, the CaAl rich sample has a micro closed-pore structure, while the CaP rich sample has a nano open-pore structure, and the CaP rich sample has smaller nanoplatelets than the CaAl rich sample.^99^ Other additives such as pore phosphate-based glass, which could be used as a sintering aid,^101^ can also influence the porosity of ceramics.

#### 3.1.3. Size of particles

Normally, it is thought that the bioactivity of calcium phosphate materials is achieved by releasing substances from the implant surface and the precipitation of a biological apatite layer. In this case, decreasing the size of the particles arguably leads to the increase in the area of the CaP surface, which could increase the dissolution of Ca and P ions, resulting in more apatite deposition and greater protein absorption, osteoblast adhesion and thus increase bone growth. Lin *et al.*^102^ found that the HA bioceramics scaffolds with the micro-/nano-topography surfaces significantly enhanced cell attachment and viability, alkaline ALP activity, and mRNA expression levels of osteogenic markers and angiogenic factors of ADSCs (Adipose Derived Stromal Cells). Coathup *et al.*^103^ suggested that the highest cell viability, the largest gene expression upregulation of two different osteogenic markers including osteocalcin and osteopontin, as well as the least disrupted cell cytoskeleton and cell morphologies were noticed for the calcium phosphate powder composed of the smallest, spherical nanosized particles. Various sizes of particles may have different effects on different stages of bone reformation. It has been reported that the speed of bone growth adjacent to larger particles (250–500 μm) was initially more rapid, while at a later stage the smallest granules (90–125 μm) induced more bone formation.^103^ According to Wang *et al.*,^104^ histological evaluation of the explants showed abundant bone in all BCP samples with particle size of 212–300 μm, 106–212 μm, and 45–106 μm, while no bone was seen in any sample having particle size smaller than 45 μm. It is most likely the particle size that affects inductive bone formation *via* macroporous structures for body fluid infiltration, cell tissue in-growth and angiogenesis.^104^ Moreover, changes in the concentration of transforming growth factor-β1 and PGE2 depend on particle size and seem to be more significant and persist for longer in smaller hydroxyapatite particle groups. Besides, calcium phosphate with the highest specific surface area and the smallest spherical particle size was found to be the most effective in both drug loading and release, consequently having the highest antibacterial efficiency,^105^ mechanical strength,^106^ (according to the well-known Hall–Patch equation) and injectibility.^107,108^ There was also a report suggesting that when the HA particle diameter increases from 13.7 nm to 14.9 nm, cell growth accordingly exhibits 1.60-fold increase.^109^ Similarly, according to Sun *et al.*,^110^ adding various sized-HA particle osteoblast cultures significantly affected cell amount, and the smallest-sized HA had the smallest cell populations. The leading reason for this kind of adverse effect of small-sized particles is perhaps that these particles take up valuable inter-granular space, which is essential for invasion by body fluid, nutrient host cells and blood vessels. For this reason, it is necessary to carry out more exhaustive studies on the optimal size of particles of CaP to maximally enhance bone growth in the future.

### 3.2. Dopants

The addition of biologically active inorganic ions into the calcium phosphate matrix to stimulate cellular reactions is a promising strategy to accelerate bone defect healing *via* the addition of various dopants inside the crystalline matrix to adjust its crystal structure and thus affect its properties.

**Fig. 7:**
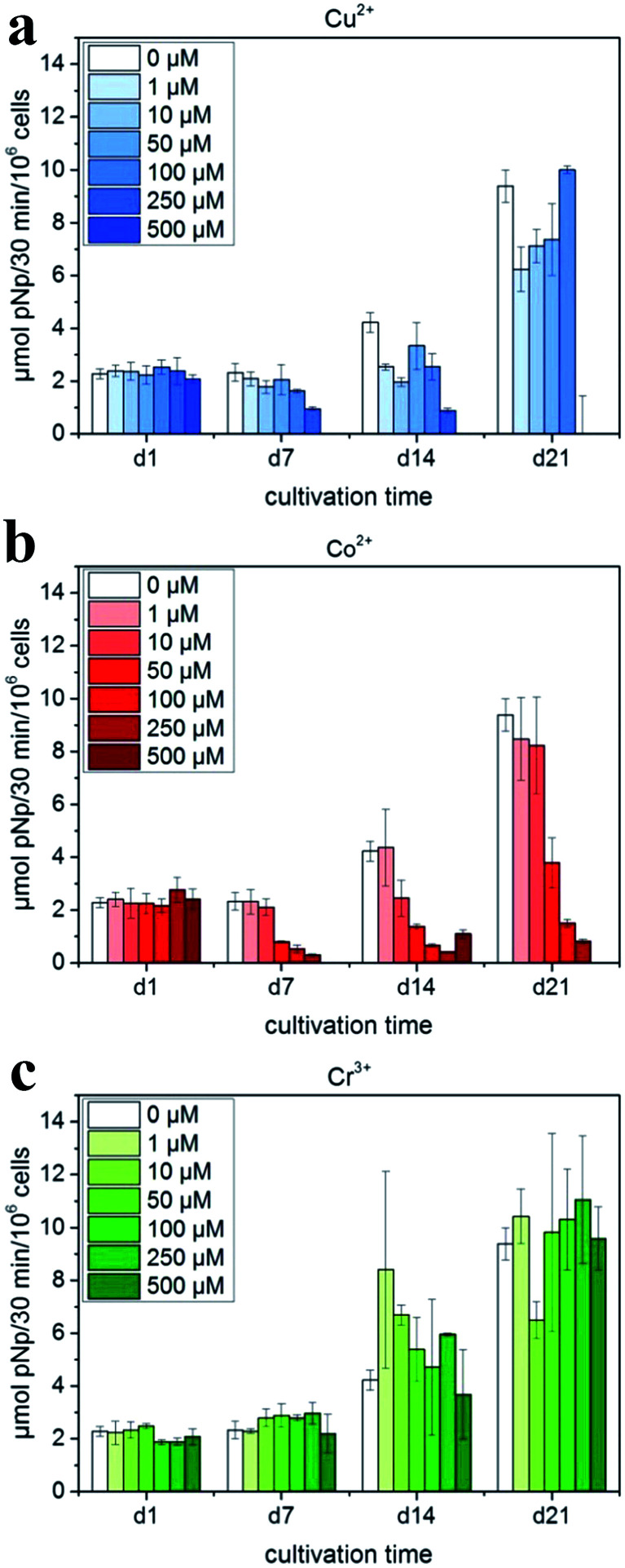
ALP activity of hMSC cultivated in the presence of Cu^2+^, Co^2+^ and Cr^3+^ ions added to cell culture medium (with OS) in various concentrations: (a) Cu^2+^: 0–500 μM (μmol L^−1^); (b) Co^2+^: 0–500 μM; and (c) Cr^3+^: 0–500 μM. Reprinted with permission from ref. 111 (Copyright © 2017, Elsevier).

According to Schamel *et al.*,^111^ the cell experiments with hMSC revealed the positive influence of the modification with 50 mmol Cr^3+^ and – to a less extent – with 10 mmol Cu^2+^ on cytocompatibility; the modification with Co^2+^ resulted in the suppression of cell growth and osteogenic differentiation (Fig. 7). However, in the experiment of Albayrak *et al.*,^112^ injecting Co-doped calcium phosphate microparticles into the intra-bone marrow of osteoporotic rats was found to increase the bone mineral density (BMD).

As many researchers have reported, doping Ag into calcium phosphate cement can enhance antibacterial properties.^113–116^ However, the incorporation of silver must be strictly controlled during the synthesis, since this influences the silver release kinetics. Range *et al.*^117^ compared two kinds of silver-containing biomaterials, namely, stoichiometric silver phosphate and silver-doped calcium phosphate; antimicrobial studies showed that both had a high bactericidal effect. However, due to a high release rate of silver ions in stoichiometric silver phosphate, it leads to a cytotoxic effect on eukaryotic cells as well, making the silver phosphate unsuitable as a silver-containing ceramic material. Interestingly, Rodriguez *et al.*^118^ found that the calcium phosphate coatings with 2.7% of selenium also resulted in a significant anti-proliferative effect (*p* < 0.01) on cancerous osteoblasts (MG63) in a preliminary study, and anti-biofilm properties (*p* < 0.01) against *Staphylococcus epidermidis* and *Staphylococcus aureus* bacterial strains. In this case, it is reasonable to believe that more studies using Se-doped calcium phosphate as prominent antibacterial materials need to be done in the future, taking cost into consideration.

The effects of Li, Fe, Mg and Zn doped in CaP on biomedical applications have also been investigated. Recent studies suggest that the presence of Fe^3+^ affects the crystallinity and solubility of HA,^119,120^ while small amounts of iron were found to have a positive impact on the biomedical properties of HA.^121,122^ Micro-CT showed better repair of bone defects in Li-doped CPC groups, compared to the blank group.^123^ Dual addition of bivalent magnesium (Mg^2+^) and cobalt (Co^2+^) ion dopants to hydroxyapatite had a significantly higher protein absorption capacity in comparison to pure hydroxyapatite.^124^ Detailed analysis pertaining to the bone cell (MG-63) compatibility and differentiation revealed that this doping process significantly promoted cell proliferation and differentiation.^124^ Apart from having a similar function, Zn ions are thought to also possess a potent and selective inhibitory effect on osteoclastic bone resorption.^125^

### 3.3. Surface modification

To endow calcium phosphate with better bioactivity, coating bioactive membranes on CaP matrix have been devised and applied to clinical treatment for a long time.

Chitosan is a type of natural biopolymer with complete biocompatibility, thus allowing it to be applied in various medical fields. The study done by Coelho *et al.*^126^ showed that the Ca–P/chitosan coated on metals could generate sufficient adhesion with substrate, more so than the pure chitosan coating. Chitosan-coated Mg–Zn–tricalcium phosphate composite was found to slow down the *in vivo* degradation of the composite after surgery, improve the concrescence of the bone tissues and play a unique role in enhancing the corrosion resistance of the implant.^127^

**Fig. 8:**
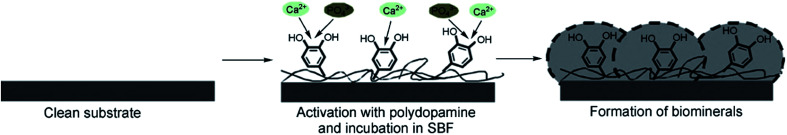
Formation mechanism of apatite deposited on the substrate with PDA coating. Reprinted with permission from ref. 128 (Copyright © 2010, John Wiley and Sons).

Due to the improvement of cell adhesion and excellent biocompatibility, polydopamine (PDA) has been widely used in the surface modification of biomaterials. For instance, Ryu *et al.*^128^ introduced dopamine (DA) solution, oxidized for two days, into CPC, and formed PDA on the surface of CPC utilizing non-oxidized DA, by which it was possible to obtain faster mineralization and formation of bone-like hydroxyapatite with nano-micro structure. The formation mechanism of apatite on the substrate with PDA coating is shown in Fig. 8.^128^

The calcium phosphate substitute covered with autogenous periosteum has the ability to provide a sufficient supply of bone tissue, osteoblasts and cell growth factors for biomaterials to promote and stimulate the formation of new bone. The bending strength test showed that the sample covered with autogenous periosteum is much better than the one without periosteum, and reached half of the strength of normal bone after six months of implanting. Histology observations also confirmed that the sample covered with autogenous periosteum exhibited a remarkably higher osteogenesis rate and osteogenesis quality than the pure CaP substitute.^54^

As one of the most abundant structural proteins in hard tissues and a well-known mediator of osteoblast cellular functions such as initial attachment, proliferation, and differentiation,^129^ collagen has been widely explored as a coating on calcium phosphate scaffolds, including unmodified/modified microporous BCP^130^ and HA.^129,131^

Although these various coatings provide the scaffold with the unique enhancement of different properties such as improved osteoinductivity and adsorption of bone morphogenetic proteins, some coating solutions used for impregnating scaffolds may lead to the considerably decreased porosity of the scaffold, due to the high viscosity, which could impact the in-growth of bone cells into the scaffold.^132^ Thus, further research is necessary to deal with the problems caused by sticky coating solutions.

### 3.4. Environmental factors

When a bone graft substitute is implanted into the body, there will be a process of interaction between the implant and the surrounding environment including body fluid, which consists of various non-organic ions (Ca^2+^, Mg^2+^, Cl^−^*etc.*), organic substances (glucose, protein, ATP *etc.*) and some gases (CO_2_, N_2_, O_2_*etc.*). After the dissolution of the surfaces of the biomaterials in this environment, the increase of substances such as Ca^2+^ and PO_4_^3−^ in body fluid will gather at the graft surface, followed by their acting as the raw materials for the formation of apatite-like phosphate, absorbing proteins and other growth factors that trigger the regeneration of new bone tissue. Besides, there are also some other factors influencing the bioactivity of bone graft, such as the temperature, the fluid flow rate, the special pressure and various growth factors.

To elucidate what effects these factors will have on an implant *in vivo* is hard to achieve because of the high requirements of techniques, expensive costs of animal tests and the long periods of experiments, *etc.* Thus, it is necessary to create a special environment, identical to body fluid, so that the whole process of the degradation of the implant can be simulated. Simulated body fluid (SBF) contains all kinds of ions whose concentrations are at the same level as those in human blood plasma. Moreover, the pH value is also adjusted to the normal level of body fluid. By investigating the influences of different factors in SBF on the performance of biomaterial, people are able to approximately estimate the real situation of implants *in vivo*.

In a large number of studies, it has been assumed that the *in vitro* apatite-forming ability measured by the simulated body fluid (SBF) test is a predictor of *in vivo* bioactivity. In the experiment done by Wolke *et al.*,^133^ the *in vitro* test suggested that all of the porous calcium phosphate coatings induced the formation of homogeneous and adherent CaP precipitation layers, and *in vivo* tests then found that all coatings became surrounded by a dense, fibrous tissue capsule after implantation. Zhang *et al.*^134^ found that increasing the concentration of Mg^2+^ in simulated body fluid (SBF) would inhibit the growth process of apatite according to the results of EDS of the composite suggesting that the Ca content in SBF increased with higher Mg^2+^ concentration (from 1× Mg to 10× Mg). This finding is in accordance with other studies reporting that magnesium ions can kinetically hinder the nucleation and growth of hydroxyapatite (HA). However, Mg may also exhibit some positive performances such as enhancing the phase stability of β-TCP when Mg is added into biphasic ceramics.^135,136^ In addition, NaCl-free or low NaCl-content 5 SBF solution led to the earlier apatite precipitation in this solution, compared to the normal 5 SBF solution. The HCO_3_^−^ content strongly affected the supersaturation and Ca–P structure by increasing the pH of the solution due to its buffering capacity. Furthermore, HCO_3_^−^ favored the attachment of CaP mineral on Ti_6_Al_4_V by decreasing the CaP crystal size, resulting in the better physical attachment of the CaP coating on the Ti_6_Al_4_V substrate.^137,138^ According to Sakaguchi *et al.*,^139^ apatite precipitation did not occur in the SBF with a low Na^+^ concentration, whereas Ca^2+^ had little effect on the initial apatite precipitation.

Few researchers have investigated the influence of the temperature of SBF on the biological behavior of materials, whereas several studies have been reported for metallic biomaterials. High temperature and high concentration of SBF can greatly accelerate the deposition and increase the size of HA formed on the surface of titanium, and even affect the morphology of deposited HA. As shown in the experiment by Li *et al.*,^140^ the diameter of the spherical HA exceeds 30 μm after soaking at 57 °C (20 °C higher than the human body), in 3 SBF for only 1 day. Since the mechanisms of apatite layer formation on metallic and CaP materials are identical, it is reasonable to deduce that the temperature of SBF may have a similar effect on CaP. The studies with results of optimal temperature for metallic implants can also provide valuable references and statistics that would be of great help for further research using SBF.

**Fig. 9:**
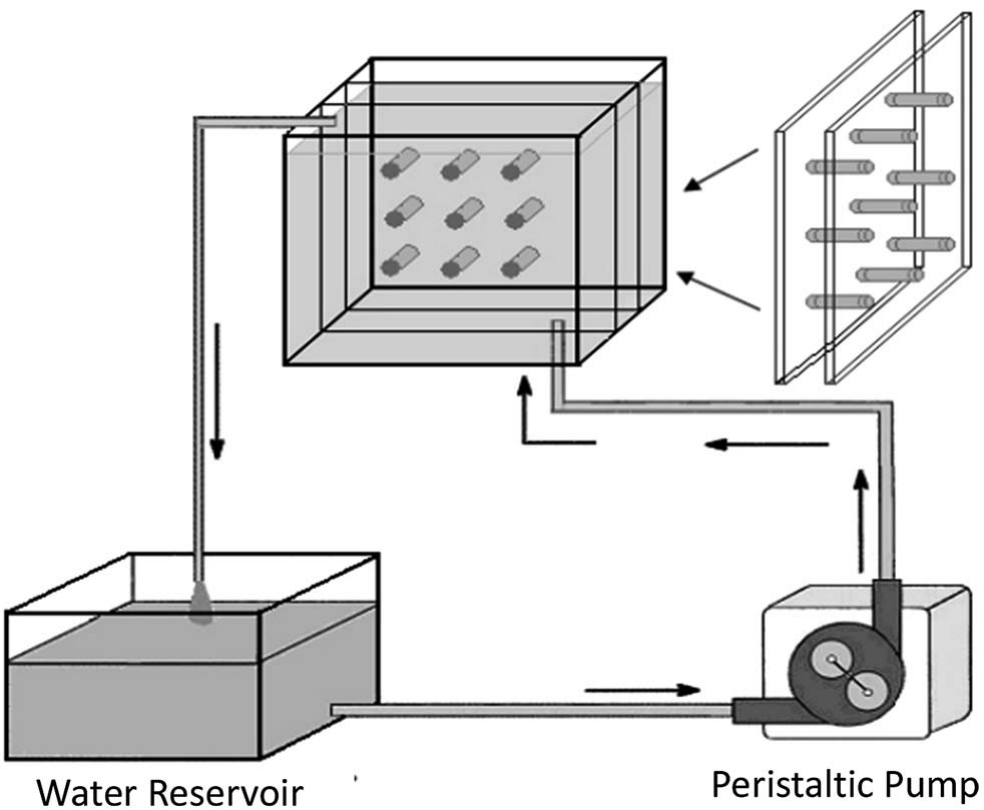
Schematic showing the apparatus for studying the degradation of scaffolds under conditions of fluid flow.^141^ Reprinted with permission from ref. 141 (Copyright © 2007, Trans Tech Publications).

Regarding the influence of SBF flow rate, Ca–P formation on aporous β-TCP/PLLA scaffold surfaces in dynamic simulated body fluid (dSBF) occurred slower than in static simulated body fluid (sSBF), and became more difficult with increasing the flow rate of dSBF. Apatite formations were analyzed based on classical crystallization theories of thermodynamics and kinetics. In sSBF, the Ca^2+^, PO_4_^3−^ from the scaffolds diffused with difficulty into sSBF solution so that the concentration of Ca and P increased to the threshold of nucleation and formed crystal nuclei. With dSBF (the schematic is shown in Fig. 9), the Ca^2+^, PO_4_^3−^ can easily diffuse into solution and be brought out of the sample chamber by the flow of SBF. Therefore, on the surface of the specimens, it was hard to achieve the threshold concentration of nucleation, but in the inner pore, the dissolved ions were hard to remove and there was easy accumulation of high Ca^2+^ and PO_4_^3−^ concentrations for nucleation. Therefore, spherical apatite individual crystals can be easily found on the walls of inner pores.^141^

Calcium phosphate biomaterials with excellent mechanical strength are widely employed in the fields of load-bearing bone repair such as joint impact and the fracture of tibia. Thus, immersing materials in SBF in conjunction with a simulation of load conditions is necessary to evaluate whether the implant will be able to simultaneously function as a bioactive scaffold and a support. Kang *et al.*^142^ examined the effects of mechanical loading on the *in vitro* degradation characteristics and kinetics of porous PLLA/β-TCP scaffolds. They found that the porosity and decrease of the compressive strength under static compressive loading were lower than that of a non-loading case, and so was the mass loss rate. This might be due to the loading retarding the penetration, absorption and transfer of simulated body fluid.

Apart from acting as a predictor for *in vivo* tests, SBF can also promote CPC mineralization and improve the bioactivity to better integrate with the host when immersing the ceramics in SBF prior to implantation. In fact, proteins, along with other organic molecules, are also active players in the regulation of the biomineralization processes *in vivo*. Huang *et al.*^9^ found that BCP pre-incubated in SBF and BSA–SBF (bovine serum albumin–simulated body fluid) up-regulated ALP activity and osteogenic related genes and proteins, thus testifying to the positive effect of SBF and BSA–SBF. Moreover, the special SBF that was prepared with the addition of BSA remarkably enhanced the cell growth.

There still are limitations to *in vitro* tests regarding the complex ingredients of SBF and possible reactions between them, and it is therefore hard to define the exact effect of each factor on the bioactivity of CaPs.

## 4. Limitations and improvements of CaP

Resorbable CaP ceramics are attractive materials for bone regeneration, but they are intrinsically brittle and thus unsuitable for use in load-bearing sites. Moreover, introducing high porosity is required to encourage better cellular in-growth into bone regeneration scaffolds and is detrimental to the mechanical strength of the material.^143^ By coating CaP ceramic on biocompatible metallic implants with high mechanical strength, an advanced composite material or incorporating CaP with polymers, excellent load-bearing performance and bioactivity can be achieved *in vivo*.

### 4.1. Coating CaP on metallic materials

Titanium and its alloys are bio-inert, having a high corrosion resistance, and sufficient mechanical strength for most indications.^144,145^ In this case, Ti is commonly employed as the substrate, coated with brittle, but bioactive CaP. Gross *et al.*^146^ found that titanium promoted the plastic deformation of the coating compared to stainless steel and Co–Cr substrate during contact nanofatigue. Kaemmerer *et al.*^144^ prepared a titanium implant with biphasic CaP coating and found that this compound exhibited great osteoinductivity and drug delivery potential as well as mechanical stability.

**Fig. 10:**
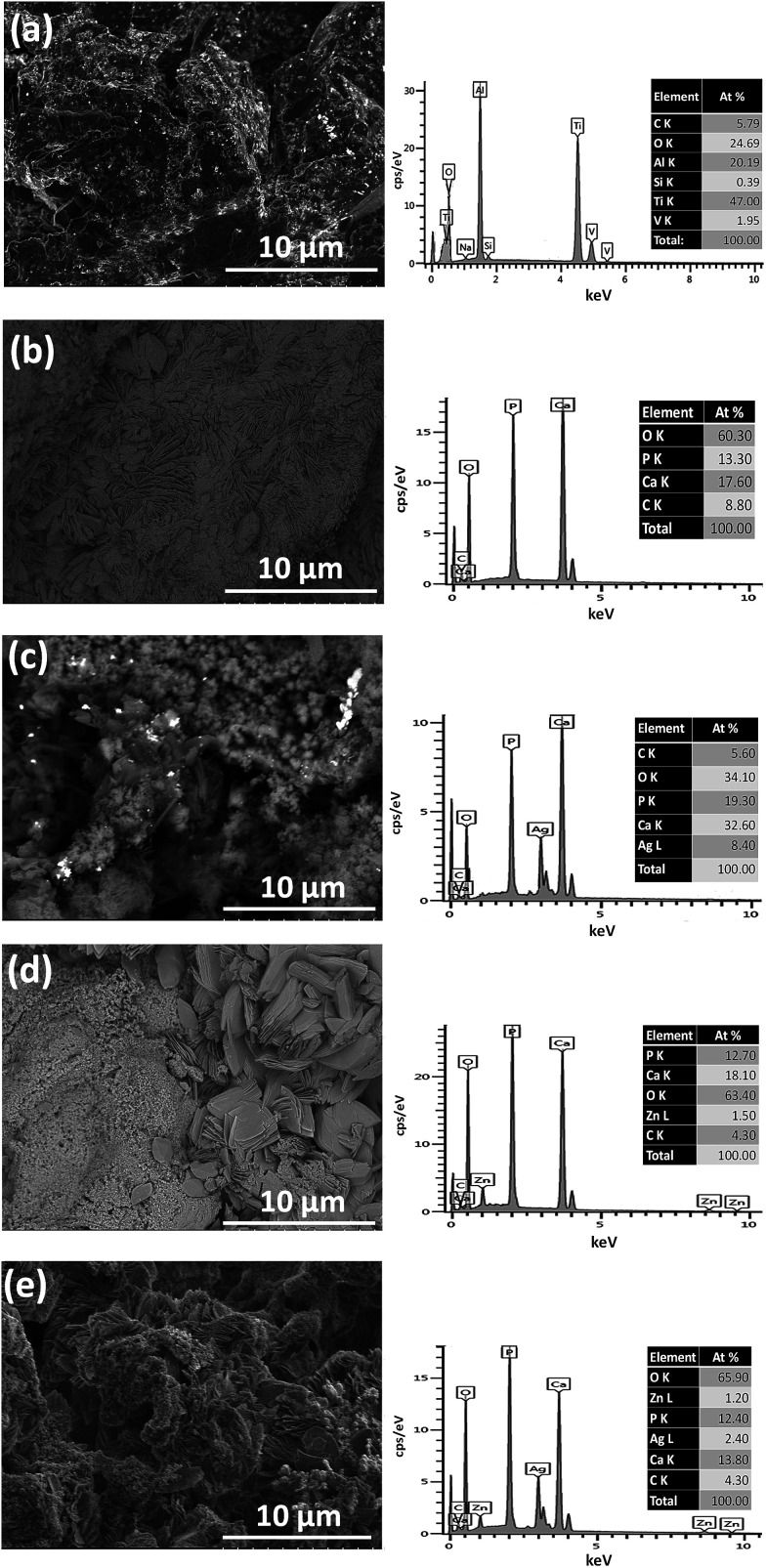
SEM and the corresponding EDX measurements on the Ti–6Al–4V substrate (a) on a CaP coating (b), on a Ag_CaP coating (c), on a Zn_CaP coating (d) as well as on a AgZn_CaP coating (e). Reprinted with permission from ref. 149 (Copyright © 2016, Elsevier).

As for the surface of the coated substitute, increasing the surface area and porosity of the implant can improve bone in-growth and the coefficient of friction between the bone and implant, thereby reducing micromotion and increasing osseointegration. An anchor-like surface topography with a secondary interconnected porous coating was prepared by a direct metal laser sintering method.^147^ This special surface modification was proved to significantly improve primary implant fixation and bone in-growth, and decreased the micromotion amplitude process in both *in vitro* and large animal *in vivo* studies. Lan *et al*.^148^ combined acid etching with UV exposure to alter the structure and surface energy. The resulting surface improved osteoblast ALP production and deposited mineralization, while decreasing the presence of *S. aureus* and *S. epidermidis* by approximately 70%. Besides, the morphology of pure calcium phosphate layers can be changed when Ag and/or Zn components are introduced into the basic electrolyte (Fig. 10).^149^ When silver and/or zinc particles are incorporated, the CaP particles become smaller and, in some cases, flake-like aggregates are formed. In addition, CaP coatings consisting of a mixture of different calcium phosphate phases such as DCP and HAp can be obtained.

Doping additives such as Ag and Zn alter the morphology of the coatings whilst promoting the antimicrobial properties and bioactivity^150^ of implant materials. However, these may lead to a loss of corrosion resistance of at least one order of magnitude.^149^ The coated surfaces doped with Ag or Zn inhibited the growth, colonization and adherence of *P. gingivalis*, resulting in the reduced thickness of biofilms and bacterial inhibition in the culture medium, as compared to the uncoated materials.^150^ Sr was added by Geng *et al.*^151^ as a binary dopant to reduce the cytotoxicity of Ag, while maintaining good antibacterial properties.

Saeed *et al.*^146^ proposed a contact nanofatigue testing method, a rapid approach, to reveal the combination strength of CaP-coated implants. It is widely known that the mechanical loading history of the coated implanted prosthesis includes (i) abrasion during insertion inside a bone cavity and (ii) dynamic loading during physical activity of the recipient. While abrasion assesses particulate generation from the coating surface, cyclic loading determines the resistance of the entire coating to cracking and delamination. Therefore, unlike conventional testing methods such as bond-strength testing, which is time-consuming, cyclic nanoindentation has been utilized to quickly assess the effect of cyclical loading on the HA coating on the titanium substrate. Results suggest that contact nanofatigue on an amorphous calcium phosphate splat offers a possible means to evaluate crack growth during cyclic loading and to compare materials with different characteristics (*i.e.* crystallinity, composition, grain size, *etc.*).

Our group has also been researching the preparation and characterization of CaP coatings on Ti or Mg alloys. For example, we prepared calcium phosphate coatings on the surface of self-designed Mg–Zn–Ca–Mn alloys *via* micro-arc oxidation technology. The SBF immersion test proved that our related biomaterials achieved excellent bioactivity, with the evidence of bone-like apatite being formed on the surfaces.^152,153^

### 4.2. Incorporating CaP with polymers

Introducing CaP into nanoscale ductile polymers is widely thought of as an attempt at mimicking the structure of natural bone, where nanocrystallites of CaP ceramic are bonded by thin collagen layers.

Generally, CaP coating is incorporated with polymer *via* coating on the polymer surface or mixing these two ingredients and then shaping using desired scaffolds. Coated composites are usually prepared by electrochemically assisted co-deposition^154^ or two-step biomimetic methods;^155,156^ the mixed composites are fabricated *via* the 3D printing technique,^157^ extrusion or direct injection into the bone defect site. Interestingly, the CaP-coated polymer nanofiber was recently fabricated by Junginger *et al.*^158^*via* the mineralization process, and it is thought to add value to the synthesis of advanced hybrid materials for bone repair.

According to Poh *et al.*,^159^ PCL scaffolds coated with CaP have a negligible effect on the scaffold's porosity and compressive Young's modulus, compared to three other groups, namely, the PCL (control), PCL/50-45S5 and PCL/50-SrBG scaffolds. However, in the study of Birgani *et al.*,^160^ CaP-coated PLA and PLA/CaP composites did not exhibit significant differences in enzymatic alkaline phosphatase activity as well as the mRNA expression of bone morphogenetic protein-2, osteopontin and osteocalcin. Thus, the method of incorporation into the hybrid material plays a less prominent role in osteogenic differentiation.

**Fig. 11:**
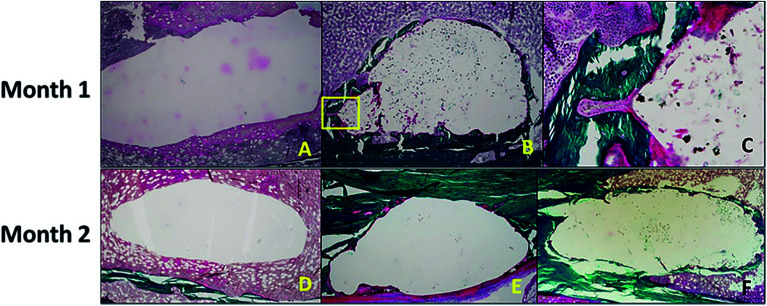
(Top) After 1 month of healing, (A) there was no bone formation around the defect in the negative control. (B and C) For the PMMA–brushite cement, osseointegration was observed with initial bone remodeling indicated by the formation of a cutting cone (area highlighted in the yellow square in (B), and magnified in (C)). (Bottom) The histology slides are shown for the cement test groups after 2 months of healing. Bone formation was only observed around the composite (E) PMMA–HA and (F) PMMA–brushite cements with no bone formation shown around (D) PMMA–K. Reprinted with permission from ref. 162 (Copyright © 2017, American Chemical Society).

Aghyarian *et al.*^161^ studied two novel composite (PMMA–CaP) bone cements including PMMA–HA and PMMA–brushite using an anatomically accurate human cadaveric vertebroplasty model. The mechanical testing included monotonic compression and cyclical fatigue tests, in which up to 57% of both PMMA–brushite and PMMA–HA reached sequence 4, demonstrating efficient reinforcement of the fractured vertebrae through stiffness restoration.^161^ In their following research,^162^ the biological performance of two PMMA–CaP cements were characterized. ALP assays showed no inhibition of osteoblast differentiation on the cement surface. Histological analysis indicated bone formation around the defect for the case of PMMA–HA and PMMA–brushite composite cements, but not for the PMMA–K cement (dark green lamellar bone was present around the defect in Fig. 11).

Except for several polymers mentioned above, PLLA was also explored by Cecen *et al.*^163^ with respect to the biocompatibility and biomechanical characteristics of loofah-based scaffolds combined with hydroxyapatite (HA), cellulose, poly-l-lactic acid (PLLA) with chondrocyte-like cells. Obvious improvements on the mechanical properties could principally be recognized in the strong interaction formed between loofah, PLLA and HA. Cells in all scaffolds produced an extracellular matrix that defined proteoglycan and type I–II collagens, suggesting that the loofah-based scaffold with desirable properties can be considered as an ideal candidate for cartilage tissue engineering applications. Similar conclusions about CaP/PLA were made by Birgani *et al.*^160^ Besides, polyelectrolyte multilayers (PEM),^164^ polyurethane (PU)^154^ and PHBV^165^ were all explored for incorporation with CaP, especially HA, in manufacturing scaffolds, and all have achieved both high mechanical strength and excellent osteointegration.

Additionally, doping some elements such as Sr^166^ into CaP coatings on polymer scaffolds is thought to be an effective measure to improve bone fixation and *in vivo* stability. It is worth mentioning that a mild annealing treatment at 130 °C for 6 h played a significant role in the fabrication process. The amorphous coatings were transformed into nanocrystalline HA films incorporating Sr (2+) with Sr/Ca molar ratios close to those of the as-deposited films. The annealing did not affect the topography and the roughness of the coatings, but did improve the hardness of the films. Recently, Zan *et al.*^164^ found that modulating the roughness of the surface of the PEMs/CaP–Col coatings can achieve optimal MSC proliferation and MSC osteogenesis, which further demonstrated that the roughness was a critical factor for bone formation.

## 5. Summary and future prospects

The most frequently used materials, biological properties and influencing factors of the biological properties of calcium phosphate, which have attracted great attention in the field of bone repair, were reviewed. As an ideal bone substitute, calcium phosphate is endowed with sufficient osteoconductivity and osteoinductivity, which ensure that sufficient body tissue forms and grows on the surface of the scaffold. Another significant property, the biodegradation of CaP, refers to various fields that are so complex that researchers have not yet come to an agreement on the mechanism. The biological properties of CaP remarkably depend on various factors. Optimal phase composition is essential to improve osteoinductivity, while the porosity is more relevant to osteoconductivity and biodegradability. Doping bioactive elements into calcium phosphate ceramics and different surface modification methods are considered as effective ways to enhance cell growth. Besides, altering the conditions of SBF can promote implant bioactivity by forming bone-like apatite on the surface. Due to the limited mechanical strength of CaP, some effective attempts including coating it on metallic materials and incorporating it with polymers have been taken to consideration, and these advanced products have been shown to achieve excellent bioactivities while maintaining mechanical stability.

The insights on several significant biological properties of CaP materials are invaluable in exploring ideal bone substitutes for employment in all sorts of clinical applications. However, some more attempts are needed to gain a better understanding of CaP and the series of reactions or biological changes after its implantation into bone defect sites. The following are some guidelines concerning the future pre-design principles and characterization methods of biological CaP materials:

(1) Apart from HA, TCP and BCP, more research is required on other CaP materials such as polycalcium phosphate.

(2) More investigations are needed on the dominant factors in CaP that stimulate cells to secrete signal molecules and how the various cytokine networks function.

(3) Other factors that influence the osteoinductivity of CaP need to be determined.

(4) More research is needed to focus on exploring both antibacterial and corrosion resistant additives added into CaP substrates.

(5) The characterization of the osteoconduction of CaP requires more reliable methods such as building numerical models concerning different variables.

(6) The biodegradation process after CaP is implanted into bone defect sites needs to be investigated on the cytobiological level.

(7) The complex correlation between osteoinduction and biodegradation is needed; for example, the possibility that degradation products may initiate the osteogenesis process should be considered.

(8) More attempts at coating polymers with CaP are required.

(9) The implantation experiments should not be done only on animals such as dogs or mice, but should also be applied to larger animals such as sheep and calves, which are considered similar to humans in terms of their environmental influences on implants.

(10) More research on the degradable performance of CaP implants under conditions of pressure and cyclic loadings are needed to simulate the environment of load-bearing bone defects.

## Conflicts of interest

There are no conflicts to declare.

## Supplementary Material

## References

[cit1] Denry I., Kuhn L. T. (2016). Dent. Mater..

[cit2] Shao R., Quan R., Zhang L., Wei X., Yang D., Xie S. (2015). J. Ceram. Soc. Jpn..

[cit3] Lee E. J., Kasper F. K., Mikos A. G. (2014). Ann. Biomed. Eng..

[cit4] Liu C., Tang R. (2014). Chin. J. Inorg. Chem..

[cit5] Tang Z., Tan Y., Ni Y., Wang J., Zhu X., Fan Y., Chen X., Yang X., Zhang X. (2017). Mater. Sci. Eng., C.

[cit6] Perez R. A., Won J., Knowles J. C., Kim H. (2013). Adv. Drug Delivery Rev..

[cit7] Yang H., Xia K., Wang T., Niu J., Song Y., Xiong Z., Zheng K., Wei S., Lu W. (2016). J. Alloys Compd..

[cit8] Yang R., Ye F., Cheng L., Wang J., Lu X., Shi Y., Fan H., Zhang X., Bu H. (2011). J. Zhejiang Univ., Sci., B.

[cit9] Huang L., Zhou B., Wu H., Zheng L., Zhao J. (2017). Mater. Sci. Eng., C.

[cit10] Yang C., Dong Y., Meng L. (2005). Chin. J. Exp. Surg..

[cit11] Duan R., Barbieri D., Luo X., Weng J., de Bruijn J. D., Yuan H. (2016). J. Orthop. Res..

[cit12] Schilling A. F., Linhart W., Filke S., Gebauer M., Schinke T., Rueger J. M., Amling M. (2004). Biomaterials.

[cit13] Chiba S., Anada T., Suzuki K., Saito K., Shiwaku Y., Miyatake N., Baba K., Imaizumi H., Hosaka M., Itoi E., Suzuki O. (2016). J. Biomed. Mater. Res., Part A.

[cit14] Cheng L., Wang T., Zhu J., Cai P. (2016). Transplant. Proc..

[cit15] Lei Y., Xu Z., Ke Q., Yin W., Chen Y., Zhang C., Guo Y. (2017). Mater. Sci. Eng., C.

[cit16] Fihri A., Len C., Varma R. S., Solhy A. (2017). Coord. Chem. Rev..

[cit17] Yu W., Wang X., Zhao J., Tang Q., Wang M., Ning X. (2015). Ceram. Int..

[cit18] Mirzaee M., Vaezi M., Palizdar Y. (2016). Mater. Sci. Eng., C.

[cit19] Ozbek Y. Y., Bastan F. E., Canikoglu N., Ozsarac U. (2016). J. Therm. Anal. Calorim..

[cit20] Ben-Arfa B. A. E., Miranda Salvado I. M., Ferreira J. M. F., Pullar R. C. (2017). Mater. Sci. Eng., C.

[cit21] Fathi M. H., Hanifi A. (2007). Mater. Lett..

[cit22] Li B., Liu Z., Yang J., Yi Z., Xiao W., Liu X., Yang X., Xu W., Liao X. (2017). Mater. Sci. Eng., C.

[cit23] Carrodeguas R. G., De Aza S. (2011). Acta Biomater..

[cit24] Kobayashi N., Hashimoto Y., Otaka A., Yamaoka T., Morita S. (2016). Materials.

[cit25] Thuermer M. B., Diehl C. E., Loureiro Dos Santos L. A. (2016). Ceram. Int..

[cit26] Jarcho M. (1986). Dent. Clin. North Am..

[cit27] Feng S., He F., Ye J. (2018). Mater. Sci. Eng., C.

[cit28] Flauder S., Gbureck U., Mueller F. A. (2014). Acta Biomater..

[cit29] Dorozhkin S. V. (2012). Acta Biomater..

[cit30] Ebrahimi M., Botelho M. G., Dorozhkin S. V. (2017). Mater. Sci. Eng., C.

[cit31] Ebrahimi M., Botelho M. G., Dorozhkin S. V. (2017). Mater. Sci. Eng., C.

[cit32] Kim K. H., Park J. Y., Park H. S., Kim K. S., Chin D. K., Cho Y. E., Kuh S. U. (2017). Yonsei Med. J..

[cit33] Tu J., Guo F., Lu C., Li B. (2011). J. Biomed. Eng..

[cit34] Weiss P., Layrolle P., Clergeau L. P., Enckel B., Pilet P., Amouriq Y., Daculsi G., Giumelli B. (2007). Biomaterials.

[cit35] Das D. , Zhang Z., Winkler T., Mour M., Guenter C. I., Morlock M. M., Machens H. and Schilling A. F., in Advances in Biochemical Engineering-Biotechnology, ed. C. Kasper, F. Witte and R. Portner, 2012, pp. 317–333 10.1007/10_2011_119 21975956

[cit36] Zhao Y., Fan J., Li Z., Liu Y., Wu Y., Liu J. (2017). Artif. Organs.

[cit37] Schnieders J., Gbureck U., Vorndran E., Schossig M., Kissel T. (2012). J. Biomed. Mater. Res., Part B.

[cit38] Wang H., Ding J., Zhu X., Fan H., Zhang X. (2010). J. Funct. Mater..

[cit39] Schnieders J., Gbureck U., Vorndran E., Schossig M., Kissel T. (2011). J. Biomed. Mater. Res., Part B.

[cit40] Albrektsson T., Johansson C. (2001). Eur. Spine J..

[cit41] Chen G., Deng C., Li Y. (2012). Int. J. Biol. Sci..

[cit42] Yi J. J., Barnes A. P., Hand R., Polleux F., Ehlers M. D. (2010). Cell.

[cit43] Tortelote G. G., Reis R. R., Mendes F. D. A., Abreu J. G. (2017). Cell. Signalling.

[cit44] Zhang H., Wang J., Deng F., Huang E., Yan Z. (2015). Biomaterials.

[cit45] Jang S., Cho H., Park J., Jeong H. (2017). Neurosci. Lett..

[cit46] Rieger M. E., Zhou B., Solomon N., Sunohara M., Li C., Cu N., Liu Y., Pan J., Minoo P., Crandall E. D., Brody S. L., Kahn M., Borok Z. (2016). J. Biol. Chem..

[cit47] Vega O. A., Lucero C. M. J., Araya H. F., Jerez S., Tapia J. C., Antonelli M., Salazar-Onfray F., Las Heras F., Thaler R., Riester S. M., Stein G. S., van Wijnen A. J., Galindo M. A. (2017). J. Cell. Biochem..

[cit48] Nalesso G., Sherwood J., Bertrand J., Pap T., Ramachandran M., De Bari C., Pitzalis C., Dell'Accio F. (2012). Int. J. Exp. Pathol..

[cit49] Stewart S., Gomez A. W., Armstrong B. E., Henner A., Stankunas K. (2014). Cell Rep..

[cit50] Okamoto M., Udagawa N., Uehara S., Maeda K., Yamashita T., Nakamichi Y., Kato H., Saito N., Minami Y., Takahashi N., Kobayashi Y. (2014). Sci. Rep..

[cit51] Weivoda M. M., Ruan M., Hachfeld C. M., Pederson L., Howe A., Davey R. A., Zajac J. D., Kobayashi Y., Williams B. O., Westendorf J. J., Khosla S., Oursler M. J. (2016). J. Bone Miner. Res..

[cit52] Jing Z., Chunlei Z., Fang X., Hong C. (2012). China Cattle Science.

[cit53] Oh K., Ko Y., Jaiswal S., Whang I. (2016). J. Mater. Sci.: Mater. Med..

[cit54] Zhang C., Wang J., Lu B., Yao Y., Fen H., Jiang X., Deng G., Liu L., Heng D., Zhang X. (2003). Med. J. Natl. Defending Forces Northwest China.

[cit55] Scheufler K. M., Diesing D. (2015). Orthopade.

[cit56] Ishack S., Mediero A., Wilder T., Ricci J. L., Cronstein B. N. (2017). J. Biomed. Mater. Res., Part B.

[cit57] Goetz W., Lenz S., Reichert C., Henkel K., Bienengraeber V., Pernicka L., Gundlach K. K. H., Gredes T., Gerber T., Gedrange T., Heinemann F. (2010). Folia Histochem. Cytobiol..

[cit58] D'Elia N. L., Gravina N., Ruso J. M., Marco-Brown J. L., Sieben J. M., Messina P. V. (2017). J. Colloid Interface Sci..

[cit59] Hashemibeni B., Dehghani L., Sadeghi F., Esfandiari E., Gorbani M., Akhavan A., Tahani S. T., Bahramian H., Goharian V. (2016). Int. J. Prev. Med..

[cit60] Fern A., Ndez J. E., Manuel Garcia-Aznar R. J., Masid M. (2017). Z. Angew. Math. Mech..

[cit61] Albrektsson T., Johansson C. (2001). Eur. Spine J..

[cit62] Yu T., Dong C., Shen Z., Chen Y., Yu B., Shi H., Zhou C., Ye J. (2016). Mater. Sci. Eng., C.

[cit63] Bouler J. M., Pilet P., Gauthier O., Verron E. (2017). Acta Biomater..

[cit64] Johari B., Ahmadzadehzarajabad M., Azami M., Kazemi M., Soleimani M., Kargozar S., Hajighasemlou S., Farajollahi M. M., Samadikuchaksaraei A. (2016). J. Biomed. Mater. Res., Part A.

[cit65] Hu J. Z., Yang Z. M., Zhou Y. C., Liu Y., Li K. Y., Lu H. B. (2015). J. Mater. Sci.: Mater. Med..

[cit66] Emilov-Velev K., Clemente-de-Arriba C., Alobera-García M. Á., Moreno-Sansalvador E. M., Campo-Loarte J. (2015). Revista Española de Cirugía Ortopédica y Traumatología.

[cit67] Barbieri D., Yuan H., Ismailoglu A., de Bruijn J. (2017). Tissue Eng., Part A.

[cit68] Carrel J., Wiskott A., Scherrer S., Durual S. (2016). Clin. Implant Dent. R..

[cit69] Frasca S., Norol F., Le Visage C., Collombet J., Letourneur D., Holy X., Sari Ali E. (2017). J. Mater. Sci.: Mater. Med..

[cit70] Bose S., Tarafder S., Bandyopadhyay A. (2017). Ann. Biomed. Eng..

[cit71] Friedmann A., Dard M., Kleber B. M., Bernimoulin J. P., Bosshardt D. D. (2009). Clin. Oral Implants Res..

[cit72] Yang C. (2001). Oral Surgery, Oral Medicine, Oral Pathology, Oral Radiology, and Endodontology.

[cit73] Schilling A. F., Filke S., Brink S., Korbmacher H., Amling M., Rueger J. M. (2006). Eur. J. Trauma.

[cit74] Tan L., Yu X., Wan P., Yang K. (2013). J. Mater. Sci. Technol..

[cit75] Sheikh Z., Najeeb S., Khurshid Z., Verma V., Rashid H., Glogauer M. (2015). Materials.

[cit76] Perrotti V., Nicholls B. M., Horton M. A., Piattelli A. (2007). J. Bone Miner. Res..

[cit77] Nakamura M., Hentunen T., Salonen J., Nagai A., Yamashita K. (2013). J. Biomed. Mater. Res., Part A.

[cit78] Ogle M. E., Segar C. E., Sridhar S., Botchwey E. A. (2016). Exp. Biol. Med..

[cit79] Ciapetti G., Di Pompo G., Avnet S., Martini D., Diez-Escudero A., Montufar E. B., Ginebra M., Baldini N. (2017). Acta Biomater..

[cit80] Neuhaus B., Tosun B., Rotan O., Frede A., Westendorf A. M., Epple M. (2016). RSC Adv..

[cit81] Winkler T., Hoenig E., Gildenhaar R., Berger G., Fritsch D., Janssen R., Morlock M. M., Schilling A. F. (2010). Acta Biomater..

[cit82] Wang J., Liu D., Guo B., Yang X., Chen X. (2017). Acta Biomater..

[cit83] Wang M., Yu Y., Dai K., Ma Z., Liu Y. (2016). Biomater. Sci..

[cit84] Zhang T., Gao J., Qu S., Li M., Weng J. (2010). Chin. J. Inorg. Chem..

[cit85] Ergun C., Liu H., Webster T. J., Olcay E., Yilmaz S., Sahin F. C. (2008). J. Biomed. Mater. Res., Part A.

[cit86] Bajpai I., Kim D. Y., Kyong-Jin J., Song I., Kim S. (2017). J. Biomed. Mater. Res., Part B.

[cit87] Wang J., Chen Y., Zhu X., Yuan T., Tan Y., Fan Y., Zhang X. (2014). J. Biomed. Mater. Res., Part A.

[cit88] Lu X. Y., Ran J. G., Gou L. (2005). Rare Met. Mater. Eng..

[cit89] Uskokovic V., Desai T. A. (2013). J. Biomed. Mater. Res., Part A.

[cit90] Chen F., Yang B., Qi C., Sun T., Jiang Y., Wu J., Chen X., Zhu Y. (2015). RSC Adv..

[cit91] Pripatnanont P., Praserttham P., Suttapreyasri S., Leepong N., Monmaturapoj N. (2016). Int. J. Oral Maxillofac. Implants.

[cit92] Baradararan S., Hamdi M., Metselaar I. H. (2012). Adv. Appl. Ceram..

[cit93] Lee J., Choi H., Yoon S., Kim B., Park H. (2013). J. Ceram. Process Res..

[cit94] Chen Z., Zhang X., Yang Y., Zhou K., Wragg N., Liu Y., Lewis M., Liu C. (2017). Ceram. Int..

[cit95] Woodard J. R., Hilldore A. J., Lan S. K., Park C. J., Morgan A. W., Eurell J. A. C., Clark S. G., Wheeler M. B., Jamison R. D., Johnson A. J. W. (2007). Biomaterials.

[cit96] Kasuya A., Sobajima S., Kinoshita M. (2012). J. Orthop. Res..

[cit97] Coathup M. J., Hing K. A., Samizadeh S., Chan O., Fang Y. S., Campion C., Buckland T., Blunn G. W. (2012). J. Biomed. Mater. Res., Part A.

[cit98] Tsukanaka M., Fujibayashi S., Otsuki B., Takemoto M., Matsuda S. (2015). J. Mater. Sci.: Mater. Med..

[cit99] Yang J., Hu X., Huang J., Chen K., Huang Z., Liu Y., Fang M., Sun X. (2016). Nanoscale.

[cit100] Kim D., Kim K., Chun H., Kim T., Park H., Yoon S. (2014). Ceram. Int..

[cit101] Yang Y., He F., Ye J. (2016). Mater. Sci. Eng., C.

[cit102] Xia L., Lin K., Jiang X., Fang B., Xu Y., Liu J., Zeng D., Zhang M., Zhang X., Chang J., Zhang Z. (2014). Biomaterials.

[cit103] Coathup M. J., Cai Q., Campion C., Buckland T., Blunn G. W. (2013). J. Biomed. Mater. Res., Part B.

[cit104] Wang L., Barbieri D., Zhou H., de Bruijn J. D., Bao C., Yuan H. (2015). J. Biomed. Mater. Res., Part A.

[cit105] Uskokovic V., Batarni S. S., Schweicher J., King A., Desai T. A. (2013). ACS Appl. Mater. Interfaces.

[cit106] Lee S., Regnault W. F., Antonucci J. M., Skrtic D. (2007). J. Biomed. Mater. Res., Part B.

[cit107] Yang J., Ye J. (2008). Bull. Chin. Ceram. Soc..

[cit108] Chernousova S., Klesing J., Soklakova N., Epple M. (2013). RSC Adv..

[cit109] Moo-Chin W., Hui-Ting C., Wei-Jen S., Hsin-Fang C., Min-Hsiung H., I-Ming H. (2015). Ceram. Int..

[cit110] Sun J. S., Liu H. C., Chang W., Li J., Lin F. H., Tai H. C. (1998). J. Biomed. Mater. Res..

[cit111] Schamel M., Bernhardt A., Quade M., Wurkner C., Gbureck U., Moseke C., Gelinsky M., Lode A. (2017). Mater. Sci. Eng., C.

[cit112] Albayrak O. (2016). Mater. Charact..

[cit113] Rau J. V., Fosca M., Graziani V., Egorov A. A., Zobkov Y. V., Fedotov A. Y., Ortenzi M., Caminiti R., Baranchikov A. E., Komlev V. S. (2016). J. Funct. Biomater..

[cit114] Costescu A., Ciobanu C. S., Iconaru S. L., Ghita R. V., Chifiriuc C. M., Marutescu L. G., Predoi D. (2013). J. Nanomater..

[cit115] Bostancioglu R. B., Peksen C., Genc H., Gurbuz M., Karel F. B., Koparal A. S., Dogan A., Kose N., Koparal A. T. (2015). Biomed. Mater..

[cit116] Ureyen M. E., Dogan A., Koparal A. S. (2012). Text. Res. J..

[cit117] Range S., Hagmeyer D., Rotan O., Sokolova V., Verheyen J., Siebers B., Epple M. (2015). RSC Adv..

[cit118] Rodriguez-Valencia C., Freixeiro P., Serra J., Ferreiros C. M., Gonzalez P., Lopez-Alvarez M. (2017). Biomed. Mater..

[cit119] Kaygili O., Dorozhkin S. V., Ates T., Al-Ghamdi A. A., Yakuphanoglu F. (2014). Ceram. Int..

[cit120] Gomes S., Kaur A., Greneche J., Nedelec J., Renaudin G. (2017). Acta Biomater..

[cit121] Li Y., Widodo J., Lim S., Ooi C. P. (2012). J. Mater. Sci..

[cit122] Iafisco M., Sandri M., Panseri S., Manuel Delgado-Lopez J., Gomez-Morales J., Tampieri A. (2013). Chem. Mater..

[cit123] Li L., Qin Y., Ma G., Li B. (2016). J. South. Med. Univ..

[cit124] Wu H., Zhang R., Li X., Ni J., Zhao C., Song Y., Wang J., Zhang S., Zheng Y., Zhang X. (2014). Prog. Nat. Sci.: Mater. Int..

[cit125] Gunawan, Sopyan I., Suryanto, Naqshbandi A. (2014). Indian J. Chem., Sect. A: Inorg., Bio-inorg., Phys., Theor. Anal. Chem..

[cit126] Coelho W. T. , Fernandes J. M., Vieira R. S., Thurmer M. B. and Santos L. A., in Materials Science Forum, ed. L. Salgado and F. Ambrozio, 2012, pp. 1181–1186

[cit127] Zhao J., Chen L., Yu K., Chen C., Dai Y., Qiao X., Yan Y. (2014). Biointerphases.

[cit128] Ryu J., Ku S. H., Lee H., Park C. B. (2010). Adv. Funct. Mater..

[cit129] Guan J., Yang J., Dai J., Qin Y., Wang Y., Guo Y., Ke Q., Zhang C. (2015). RSC Adv..

[cit130] Lee M., You C., Kim K. (2015). Materials.

[cit131] Kumar M. R., Freund M. S. (2015). RSC Adv..

[cit132] Zairani N. A. S., Jaafar M., Ahmad N., Razak K. A. (2016). Ceram. Int..

[cit133] Lanao R. P. F., Hoekstra J. W. M., Wolke J. G. C., Leeuwenburgh S. C. G., Plachokova A. S., Boerman O. C., Beucken J. J. J. P., Jansen J. A. (2014). J. Tissue Eng. Regener. Med..

[cit134] Zhang J., Dai C., Wei J., Wen Z., Zhang S., Lin L. (2013). Appl. Surf. Sci..

[cit135] Kannan S., Lemos I., Rocha J., Ferreira J. (2005). J. Solid State Chem..

[cit136] Salma-Ancane K., Stipniece L., Putnins A., Berzina-Cimdina L. (2015). Ceram. Int..

[cit137] Pan H., Zhao X., Darvell B. W., Lu W. W. (2010). Acta Biomater..

[cit138] Jalota S., Bhaduri S. B., Tas A. C. (2006). J. Mater. Sci.: Mater. Med..

[cit139] Sakaguchi A., Nakano M., Hieda J., Ohtake N., Akasaka H. (2015). Appl. Surf. Sci..

[cit140] Li N., Xiao G., Liu B., Wang Z., Zhu R., Lu Y. (2016). Surf. Coat. Technol..

[cit141] Yunqing K., Guangfu Y., Kefeng W., Lin L., Li L., Yadong Y. (2007). Key Eng. Mater..

[cit142] Kang Y. , Yin G., Luo L., Wang K. and Zhang Y., in Key Engineering Materials, ed. Y. H. Kim, C. S. Cho, I. K. Kang, S. Y. Kim and O. H. Kwon, 2007, p. 273

[cit143] Gotman I. , Swain S. K., Sharipova A. and Gutmanas E. Y., in AIP Conference Proceedings, ed. V. E. Panin, S. G. Psakhie and V. M. Fomin, 2016

[cit144] Kaemmerer T. A., Palarie V., Schiegnitz E., Topalo V., Schroeter A., Al-Nawas B., Kaemmerer P. W. (2017). J. Oral Pathol. Med..

[cit145] Krzakala A., Kazek-Kesik A., Simka W. (2013). RSC Adv..

[cit146] Saber-Samandari S., Gross K. A. (2013). Acta Biomater..

[cit147] Raphel J., Holodniy M., Goodman S. B., Heilshorn S. C. (2016). Biomaterials.

[cit148] Lan G., Li M., Tan Y., Li L., Yang X., Ma L., Yin Q., Xia H., Zhang Y., Tan G., Ning C. (2015). J. Mater. Sci. Technol..

[cit149] Furko M., Jiang Y., Wilkins T., Balazsi C. (2016). Ceram. Int..

[cit150] Aranya A. K., Pushalkar S., Zhao M., LeGeros R. Z., Zhang Y., Saxena D. (2017). J. Biomed. Mater. Res., Part A.

[cit151] Geng Z., Wang R., Zhuo X., Li Z., Huang Y., Ma L., Cui Z., Zhu S., Liang Y., Liu Y., Bao H., Li X., Huo Q., Liu Z., Yang X. (2017). Mater. Sci. Eng., C.

[cit152] Dou J., You Q., Gu G., Chen C., Zhang X. (2016). Biointerphases.

[cit153] Dou J., Gu G., Chen C. (2017). Mater. Lett..

[cit154] Meskinfam M., Bertoldi S., Albanese N., Cerri A., Tanzi M. C., Imani R., Baheiraei N., Farokhi M., Fare S. (2018). Mater. Sci. Eng., C.

[cit155] Zhao X., Li H., Xu Z., Li K., Cao S., Jiang G. (2017). Surf. Coat. Technol..

[cit156] Mai T., Boye S., Yuan J., Voelkel A., Graewert M., Guenter C., Lederer A., Taubert A. (2015). RSC Adv..

[cit157] Vella J. B., Trombetta R. P., Hoffman M. D., Inzana J., Awad H., Benoit D. S. W. (2017). J. Biomed. Mater. Res., Part A.

[cit158] Junginger M., Kuebel C., Schacher F. H., Mueller A. H. E., Taubert A. (2013). RSC Adv..

[cit159] Poh P. S. P., Hutmacher D. W., Holzapfel B. M., Solanki A. K., Stevens M. M., Woodruff M. A. (2016). Acta Biomater..

[cit160] Birgani Z. T., van Blitterswijk C. A., Habibovic P. (2016). J. Mater. Sci.: Mater. Med..

[cit161] Aghyarian S., Hu X., Haddas R., Lieberman I. H., Kosmopoulos V., Kim H. K. W., Rodrigues D. C. (2017). J. Orthop. Res..

[cit162] Aghyarian S., Bentley E., Hoang T. N., Gindri I. M., Kosrnopoulos V., Kim H. K. W., Rodrigues D. C. (2017). ACS Biomater. Sci. Eng..

[cit163] Cecen B., Kozaci L. D., Yuksel M., Ustun O., Ergur B. U., Havitcioglu H. (2016). Mater. Sci. Eng., C.

[cit164] Zan X., Sitasuwan P., Feng S., Wang Q. (2016). Langmuir.

[cit165] Rasoga O., Sima L., Chiritoiu M., Popescu-Pelin G., Fufa O., Grumezescu V., Socol M., Stanculescu A., Zgura I., Socol G. (2017). Appl. Surf. Sci..

[cit166] Bianchi M., Degli Esposti L., Ballardini A., Liscio F., Berni M., Gambardella A., Leeuwenburgh S. C. G., Sprio S., Tampieri A., Iafisco M. (2017). Surf. Coat. Technol..

